# The Study of Deep Level Traps and Their Influence on Current Characteristics of InP/InGaAs Heterostructures

**DOI:** 10.3390/nano9081141

**Published:** 2019-08-09

**Authors:** Xiaohong Zhao, Hongliang Lu, Manli Zhao, Yuming Zhang, Yimen Zhang

**Affiliations:** The State Key Discipline Laboratory of Wide Band Gap Semiconductor Technology, School of Microelectronics, Xidian University, Xi’an 710071, China

**Keywords:** proton irradiation, deep level traps, recombination current, InP/InGaAs heterostructure

## Abstract

The damage mechanism of proton irradiation in InP/InGaAs heterostructures was studied. The deep level traps were investigated in detail by deep level transient spectroscopy (DLTS), capacitance–voltage (C–V) measurements and SRIM (the stopping and range of ions in matter, Monte Carlo code) simulation for non-irradiated and 3 MeV proton-irradiated samples at a fluence of 5 × 10^12^ p/cm^2^. Compared with non-irradiated samples, a new electron trap at *E*_C_-0.37 eV was measured by DLTS in post-irradiated samples and was found to be closer to the center of the forbidden band. The trap concentration in bulk, the interface trap charge density and the electron capture cross-section were 4 × 10^15^ cm^−3^, 1.8 × 10^12^ cm^−2^, and 9.61 × 10^−15^ cm^2^, respectively. The deep level trap, acting as a recombination center, resulted in a large recombination current at a lower forward bias and made the forward current increase in InP/InGaAs heterostructures for post-irradiated samples. When the deep level trap parameters were added into the technology computer-aided design (TCAD) simulation tool, the simulation results matched the current–voltage measurements data well, which verifies the validity of the damage mechanism of proton irradiation.

## 1. Introduction

The InP/InGaAs material system has the advantages of high electron mobility and large hetero-junction offsets, which promote the realization of InP-based heterojunction bipolar transistors (HBTs) in high-speed analog circuits [1]. In space communication systems, the degradations induced by electron [2], neutron [3] and proton irradiation would cause issues of reliability.

Many works have studied the electrical characteristics of InP/InGaAs devices before and after irradiation. In [4], the authors showed that the degradation of current gain and cut-off frequency were caused by proton irradiation in InP HBTs. The increase in offset voltage was induced by neutron irradiation in InP single heterojunction bipolar transistors (SHBTs) [5]. Such degradations of devices were mainly attributed to the defects in InP/InGaAs heterostructures when the devices work in extremely complex spatial irradiation environments [6]. To predict proton induced degradation of InP/InGaAs heterostructures, the nonionizing energy loss model (NIEL) was proposed [7]. All of these previous results have considerably helped with understanding the process of the irradiation damage. However, the proprieties of deep level traps induced by proton irradiation in InP/InGaAs heterostructures have not been reported to date. Therefore, in order to study defects induced by proton irradiation and discuss the recombination current mechanisms of InP/InGaAs heterostructures, it is necessary to study the properties of the traps in InP/InGaAs heterostructures. Deep level transient spectroscopy (DLTS) can conveniently detect the trap positions in the band gap, capture cross-sections, trap types and concentrations.

In this paper, the deep level traps in InP/InGaAs heterostructures are characterized by DLTS, capacitance–voltage (C–V) measurements and SRIM (the stopping and range of ions in matter, Monte Carlo code) simulation for non-irradiated and post-irradiated samples with 3 MeV protons at a fluence of 5 × 10^12^ p/cm^2^. The trap parameters were inputted into TCAD to simulate the current characteristics. The traps are responsible for the increase in recombination current and the ideality factor after irradiation in InP/InGaAs heterostructures.

## 2. Experiments 

**Figure 1:**
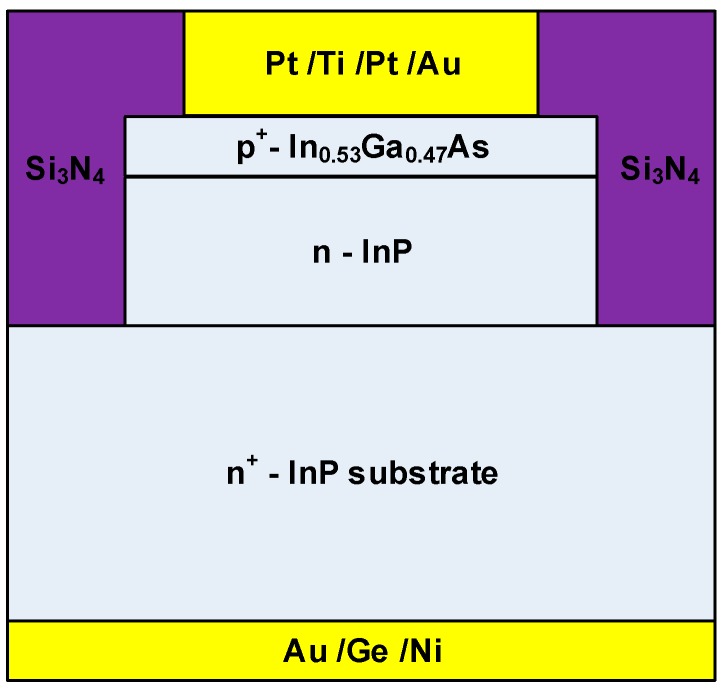
Schematic diagram of the InP/In_0.53_Ga_0.47_As heterostructure.

The device used in this study has a p^+^-In_0.53_Ga_0.47_As/n-InP heterostructure, which was grown by a V90 GSMBE system (Veeco, New York, NY, USA) in the Shanghai Institute of Microsystem and Information Technology (SMIT). The arsenic and phosphorus beams were from thermal cracking of arsine (AsH_3_) and phosphine (PH_3_) at a temperature of 410 °C. Elemental gallium (Ga) and indium (In) were used as the group-III sources. Silicon (Si) and CBr_4_ (C) were used as n-type and p-type dopants, respectively. Figure 1 shows the schematic diagram of the p^+^-In_0.53_Ga_0.47_As/n-InP heterostructure, which consists of the substrate of n-type InP (S: 1 × 10^18^ cm^−3^), a 300 nm n-InP (Si: 2 × 10^17^ cm^−3^), and a 80 nm p^+^-In_0.53_Ga_0.47_As layer (C: 3 × 10^19^ cm^−3^). Si_3_N_4_ was used for passivation. Pt/Ti/Pt/Au and Au/Ge/Ni were deposited by electron beam evaporation and then alloyed for p- and n-type ohmic contacts, respectively. 

Prior to irradiation, the characteristics of current–voltage (I–V) and capacitance–voltage (C–V) were measured at room temperature. The DLTS measurements were carried out using a HERA-DLTS system (FT 1030 1 MHz, PhysTech, PhysTech GmbH Am Mühlbachbogen 55d D—85368 Moosburg, Germany) in deep level transient Fourier spectroscopy (DLTFS, PhysTech GmbH Am Mühlbachbogen 55d D—85368 Moosburg, Germany) test mode. Compared with the conventional DLTS test, the transient amplitude could be calculated at any temperature using a Fourier transformation, and the DLTS spectra were obtained by direct DLTFS and temperature scan maximum analysis. The test temperatures were set from 30 K to 300 K.

The samples without bias were irradiated by 3 MeV protons at room temperature with a proton accelerator EN2 × 6 (High Voltage Engineering Europa B.V., Amsterdamseweg 63 3812 RR Amersfoort, Netherlands) in Peking University. The proton fluence, beam density and total irradiation time were 5 × 10^12^ p/cm^2^, 0.027 nA/cm^2^ s, and about 1 h, respectively.

## 3. Results and Discussion

### 3.1. C–V and DLTS Measurements

**Figure 2:**
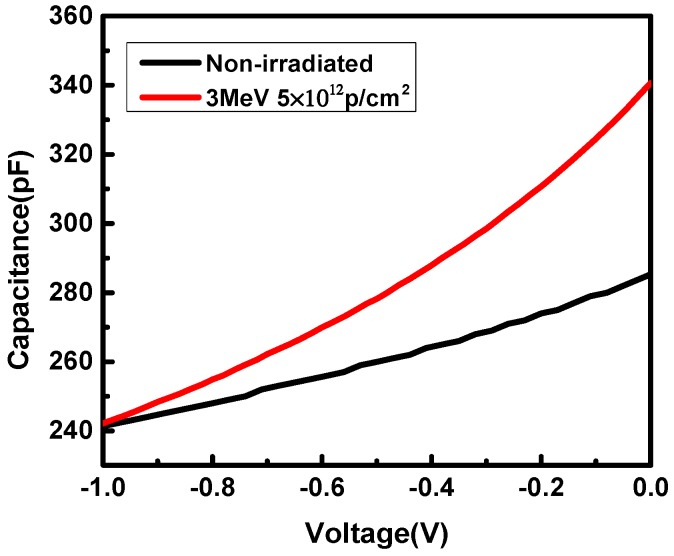
Capacitance–voltage curves.

**Figure 3:**
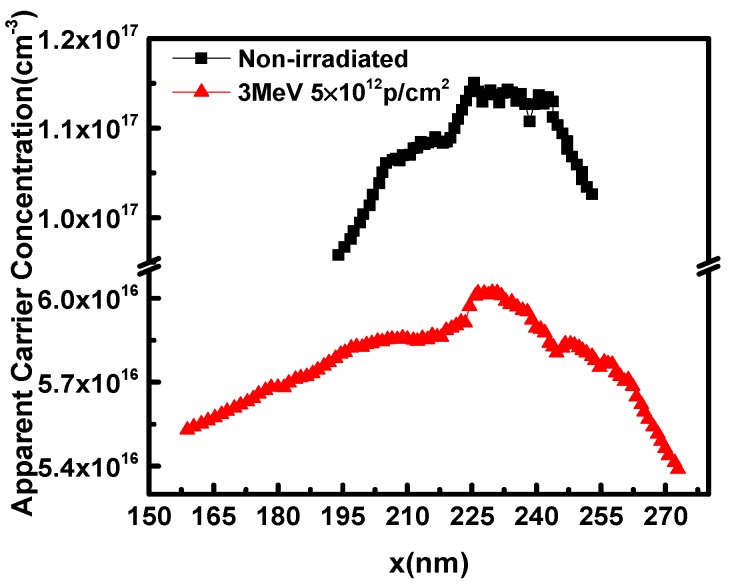
The apparent carrier concentration profile.

The C–V measurements were performed at 1 MHz to study the effects of proton irradiation on junction capacitance. Figure 2 shows the results of C–V measurements in non-irradiated and post-irradiated samples at 300 K. The differences in junction capacitance between non-irradiated and post-irradiated samples indicate that there are different amounts of interface traps in InP/InGaAs heterostructures, which might be caused by irradiation or the fabrication process of the hetero-interface. A method of calculating the interface trap charge density *σ*_i_ was proposed by Kroemer et al. [8,9,10], which can be expressed by Equation (1).

(1) $$\sigma_{i}=-\int_{0}^{\infty }\left[N_{D}\left(x\right)-n\left(x\right)\right]dx$$

where *N_D_* (*x*) is the impurity concentration distribution and *n*(*x*) is the apparent majority carrier concentration at position *x*, which is derived from the C–V profile according to the relation

(2) $$n\left(x\right)=\frac{2}{q\varepsilon_{r}\varepsilon_{0}}{\left[-\frac{d}{dV}\left(\frac{1}{C^{2}}\right)\right]}^{-1}$$

where

(3) $$x=\frac{\varepsilon_{r}\varepsilon_{0}}{C}$$

where *x* is the width of the depletion layer, which is essentially the distance of the depletion layer edge from the p–n junction, *V* is the reverse bias voltage, *C* is the capacitance per unit area, *Ɛ*_r_ is the relative dielectric constant of the material of the lightly doping side, and *Ɛ*_0_ is the vacuum permittivity. Thus, the apparent carrier concentration distribution *n*(*x*) could be obtained from Equations (2) and (3). As shown in Figure 3, the apparent carrier concentration decreases after irradiation and the maximum value is reduced to 5 × 10^16^ cm^−3^, which suggests that more interface traps are generated in post-irradiated samples. Therefore, from Equation (1), the interface trap charge densities of the non-irradiated and post-irradiated samples are *σ*_i_ = 5 × 10^11^ cm^−2^ and *σ*_i_ = 1.8 × 10^12^ cm^−2^, respectively.

**Figure 4:**
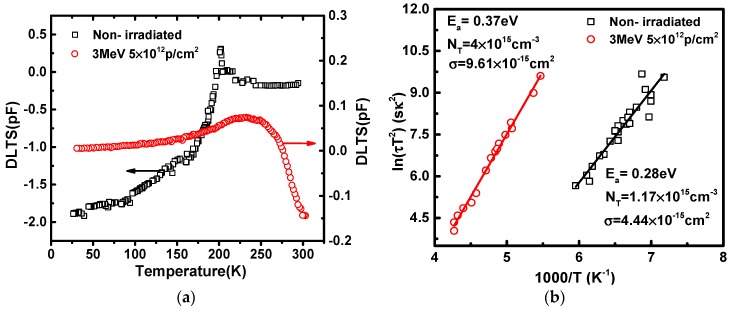
The measurement results before and after 3 MeV proton irradiation in InP/InGaAs heterostructures. (**a**) DLTS spectra and (**b**) The Arrhenius plot for deep level traps.

DLTS provides a convenient way to obtain the proprieties of deep level traps, such as trap types, their positions, concentrations, and capture cross-section. Temperature-scan DLTS at a fixed frequency of 1 MHz were used to study the deep level traps in InP/InGaAs heterostructures, with the test condition of reverse bias voltage *V*_R_ = −2 V, the filling pulse time width *t*_p_ = 1 ms and a pulse voltage *V*_p_ = 0 V to both non-irradiated and post-irradiated samples. At the same time, the DLTS data were acquired digitally for all transient data as a function of scanning temperature cooling from 300 K to 30 K and then heating from 30 K to 300 K in order to reduce the jitter error of the equipment system. Figure 4a shows the typical DLTS spectra measured in InP/InGaAs heterostructures. For non-irradiated samples, a positive peak of the DLTS spectrum appears at the temperature of about 200 K, which indicates the existence of an electron trap [11]. There is a hump near 150 K, but no trap is detected, which is due to the low density of electrically active defects [12]. While for post-irradiated samples, a new electron trap is detected at the temperature of about 235 K. 

Then, the relationship between the emission rate *e_n_* and the temperature is given in Equation (4) [11].

(4) $$e_{n}=\frac{1}{\tau_{e}}=\sigma_{n}v_{th}N_{C}\exp\left(-\frac{E_{C}-E_{T}}{kT}\right)$$

where *e_n_* is the electron emission coefficient, *τ_e_* is the emission time constant, *σ_n_* is the capture cross-section of the electron, *v_th_* is the thermal velocity, *N*_C_ is the state density in the conduction band, *E_C_* is the bottom of the condition band, *E_T_* is the trap level, *k* is the Boltzmann constant and *T* is the temperature. If Equation (4) is re-arranged, one obtains the Arrhenius presentation in Equation (5)

(5) $$\log\left(T^{2}\tau_{e}\right)=\frac{E_{C}-E_{T}}{1000k}\left(\frac{1000}{T}\right)+\log\left(\frac{1}{\gamma_{n}\sigma_{n}}\right)$$

where *γ* is a collection of constants that depend on the effective mass of the carrier and properties of the band gap. The activation energy (*E*_a_ = *E*_C_ − *E*_T_) and electron capture cross-section (*σ*_n_) could be determined from the slope and intercept, respectively.

The trap concentration parameter *N*_T_ could be obtained by the height of the peaks on the DLTS spectrum,

(6) $$\frac{\Delta C_{0}}{C\left(\infty \right)}\approx \frac{N_{T}}{2N_{d}}$$

where Δ*C*_0_ is the total amplitude of the transient, *C*(*∞*) is the steady-state capacitance, and *N_d_* is the doping concentration. 

The Arrhenius plots could be obtained from Equations (4)–(6). Figure 4b presents the Arrhenius plots for the detected traps in InP/InGaAs heterostructures, which were obtained by using different Fourier coefficients of the numerically filtered transient signal [13]. The activation energy (*E*_a_), trap concentration (*N*_T_) and electron capture cross-section (*σ*_n_) could be calculated from these coefficients. As could be clearly seen from Arrhenius plots, the electron trap positions are at *E*_C_-0.28 eV and *E*_C_-0.37 eV respectively for non-irradiated and post-irradiated samples, the concentrations of these two traps are 1.17 × 10^15^ cm^−3^ and 4 × 10^15^ cm^−3^, and the electron capture cross-sections are *σ*_n_ = 4.44 × 10^−15^ cm^2^ and *σ*_n_ = 9.61 × 10^−15^ cm^2^, respectively.

The same electron trap (*E*_C_-0.28 eV) was detected in the bulk n-InP in [14]. The damage might be caused by ion implantation in the InP material during the fabrication process, and in this case, the sharp DTLS spectra might be caused by isolated point defects. However, for 3 MeV proton energy with 5 × 10^12^ p/cm^2^ fluence, the electron traps (*E*_C_-0.28 eV) disappear. This is because the traps at *E*_C_-0.28 eV are filled with carriers induced by the proton ionization effect, which means) of the new trap is closer to the center of the energy band gap, which would have a more serious influence on the characteristics of InP/InGaAs heterostructures.

### 3.2. The Simulation of Proton Irradiation Damage by SRIM

**Figure 5:**
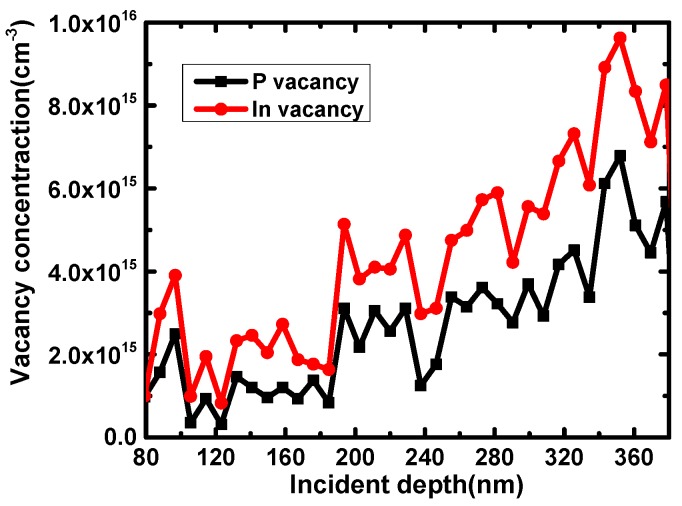
In vacancies and P vacancies in InP/InGaAs heterostructures with 3 MeV proton irradiation at the fluence of 5 × 10^12^ p/cm^2^.

The proton irradiation damage in InP/InGaAs heterostructures was simulated by SRIM. After proton irradiation, the In and P atoms displaced from their lattice sites, resulting in defect centers. Figure 5 shows the vacancy concentrations in the n-InP layer with 3 MeV proton irradiation at the fluence of 5 × 10^12^ p/cm^2^. The oscillation is caused by the calculation of random processes in SRIM; however, the trend generally increases with increasing incident depth. The densities of In vacancies are obviously higher than P vacancies and the average concentration of the In vacancy is about 4.18 × 10^15^ cm^−3^, which is close to the DLTS measurements. The In vacancy causes acceptor-like defects in the InGaAs/InP layer heterostructure according to [15], which also corresponds to the electron traps measured by DLTS. Both the DLTS measurement and the SRIM simulation results represent the trap concentrations in the bulk region, while the C–V analysis shows the trap densities at the interface. The results are summarized in Table 1. The influence of traps on current characteristics is discussed below.

### 3.3. The Effect of Deep Level Traps on Current Characteristics

The empirical form of the current–voltage relationship of the pn junction is shown in Equation (7) [16]. By taking logarithms of both sides of Equation (7), the linear curves of ln(*J*_F_)~*V* are given in Equation (8),

(7) $$J_{F}=J_{0}\exp\left(\frac{qV}{\eta kT}\right)$$

(8) $$\ln(J_{F})=\ln(J_{0})+\frac{q}{\eta kT}V$$

where *J_F_* is forward current, *J*_0_ is reverse saturation current, *q* is the electronic charge, *V* is the applied voltage, *k* is the Boltzmann constant, *T* is the temperature, and *η*, the ideality factor, equals 2 when the recombination current in the space charge region dominates [7], and *η* equals 1 when the diffusion current primarily contributes towards *J*_F_. When both currents are comparable, *η* has a value between 1 and 2.

**Figure 6:**
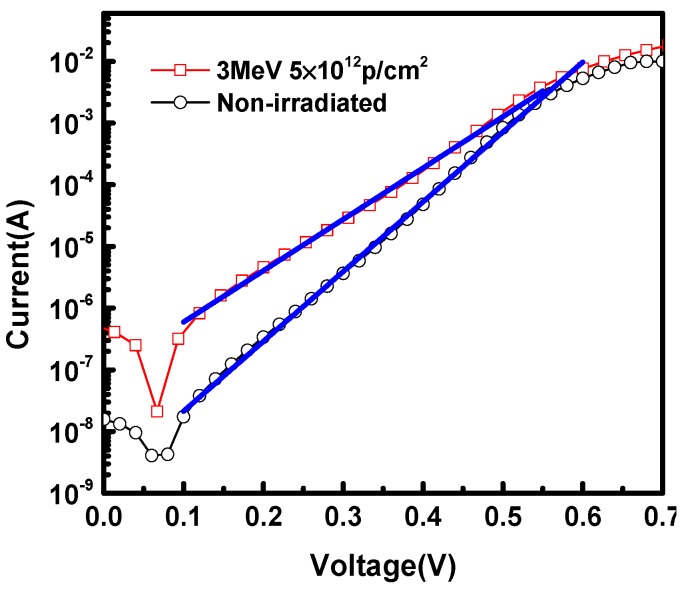
The semilog plot of I–V measurements without and with 3 MeV proton irradiation at the fluence of 5 × 10^12^ p/cm^2^ at 300 K.

Figure 6 shows the semilog plot of the I–V measurements without and after 3 MeV proton irradiation at the fluence of 5 × 10^12^ p/cm^2^ at 300 K. It is noted that forward current increases significantly after irradiation. Especially in the lower bias, the change of current is more obvious. Similar behavior was reported in [4], which investigated the base-emitter (BE) junction of InP/InGaAs HBTs under the same irradiation condition. The ideality factors (*η*) could be extracted from the slope of ln(*J*_F_)~*V* curves in Figure 6, which was 1.4 for non-irradiated samples and became 2 after 3 MeV proton irradiation at 5 × 10^12^ p/cm^2^ fluence. This indicates that the deep level trap, acting as a recombination center, results in a large recombination current at lower forward bias and makes forward current increase in InP/InGaAs heterostructures. For non-irradiated samples, the traps stemmed from the process technology of the hetero-interface. For irradiated samples, the traps were mainly induced by displacement damage caused by the collision between protons and material atoms. The parameters of deep level traps were obtained by C–V and DLTS measurements (Section 3.1). 

**Figure 7:**
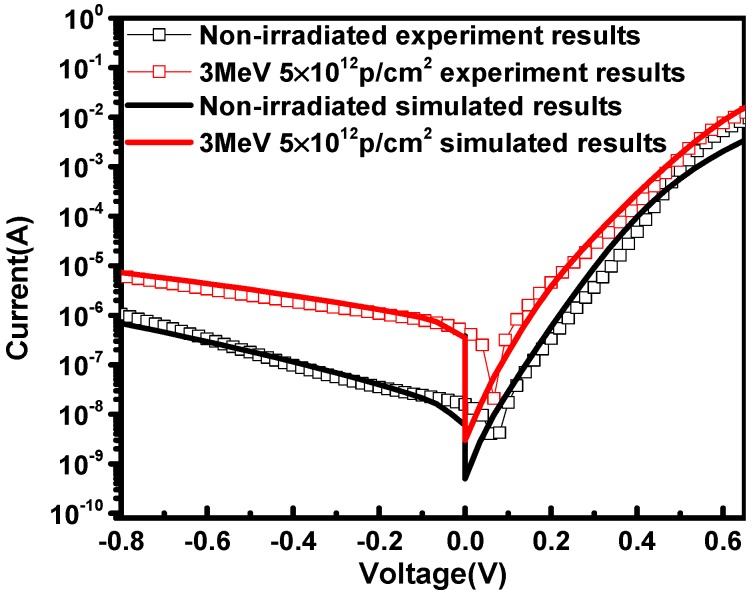
The experimental and simulated I–V curves before irradiation and after 3 MeV proton irradiation in InP/InGaAs heterostructures.

Furthermore, the current characteristics of InP/InGaAs heterostructures were simulated by putting the deep level trap parameters into the TCAD tool. Figure 7 shows the simulation and experimental results of I–V plots at 300 K before and after irradiation. The simulation results matched the experimental results well, which indicates that for post-irradiated samples, the deep level traps with an energy level at *E*_C_-0.37 eV induced by proton irradiation are responsible for the increasing recombination current at lower forward bias in InP/InGaAs heterostructures. Therefore, deep level traps acting as recombination centers have a great influence on the transport of carriers.

## 4. Conclusions

The damage mechanism of proton irradiation in InP/InGaAs heterostructures was studied. The deep level traps were analyzed in detail by DLTS, C–V measurements and SRIM simulation for non-irradiated and 3 MeV proton irradiation at the fluence of 5 × 10^12^ p/cm^2^. For non-irradiated and post-irradiated samples, the trap energy positions were at *E*_C_-0.28 eV and *E*_C_-0.37 eV respectively, the trap concentrations (*N*_T_) in bulk of these two traps were 1.17 × 10^15^ cm^−3^ and 4 × 10^15^ cm^−3^, the electron capture cross-sections (*σ*) were *σ*_n_ = 4.44 × 10^−15^ cm^2^ and *σ*_n_ = 9.61 × 10^−15^ cm^2^, and the interface trap charge densities were *σ*_i_ = 5 × 10^11^ cm^−2^ and *σ*_i_ = 1.8 × 10^12^ cm^−2^, respectively. Compared with non-irradiated samples, a new electron trap was measured by DLTS for the post-irradiated samples and was closer to the center of the forbidden band. The SRIM simulation results revealed that the trap types are mainly associated with Indium vacancies, as the concentration of them coincided with the DLTS measurements in the n-InP bulk. The deep level trap acting as a recombination center results in a large recombination current at a lower forward bias and makes forward current increase in InP/InGaAs heterostructures. After adding the deep level trap parameters into TCAD simulation tools, the simulation results matched the current–voltage measurements results well, which verifies the validity of the damage mechanism of proton irradiation.

## Figures and Tables

**Table 1:** The traps in InP/InGaAs heterostructures obtained by DLTS measurement, C–V analysis, and SRIM simulation.

Proton Irradiation	DLTS			C–V	SRIM
3 MeV 5 × 10^12^ p/cm^2^	***E*_a_**	***σ***	***N*_T_**	***σ*_i_**	**Vacancy**
	0.37 eV	9.61 × 10^−15^ cm^2^	4 × 10^15^ cm^−3^	1.8 × 10^12^ cm^−2^	4.18 × 10^15^ cm^−3^
